# EHMTI-0047. Genetic association and gene expression studies suggest that genetic variants in the SYNE1 and TNF genes are related to menstrual migraine

**DOI:** 10.1186/1129-2377-15-S1-B11

**Published:** 2014-09-18

**Authors:** L Griffiths, A Rodriguez-Acevedo, RA Smith, B Roy, HG Sutherland, RA Lea, A Frith, A MacGregor

**Affiliations:** 1 QUT, Institute of Health and Biomedical Innovation, Brisbane, Australia; 2 Clinithink Limited, Bridgend, UK

## Background

This study investigated genetic variants potentially related to menstrual migraine (MM), specifically undertaking genotyping and mRNA expression analysis of the ESR1, PGR, SYNE1 and TNF genes in MM cases and non-migraine controls.

## Methods

Migraine diagnosis was in accordance with ICHD-II. Diagnosis of pure menstrual migraine and menstrually-related migraine was confirmed by diary evidence from at least three menstrual cycles. Controls were women with no personal or family history of migraine, age and ethnicity matched to cases, where possible. A total of 37 variants distributed across14 genes were genotyped in 437 DNA samples (282 cases and 155 controls [median age 45.0 vs. 39.5 years]). In addition levels of gene expression were determined in 74 cDNA samples (41 cases and 33 controls [median age 42.0 vs. 35.5 years]). Association and correlation analysis were performed using Plink and RStudio.

## Results

SNPs rs3093664 and rs9371601 in SYNE1 and TNF genes respectively, were significantly associated with migraine in the MM population (p= 0.008; p=0.009 respectively). Analysis of qPCR results found no significant difference in levels of gene expression between cases and controls. However, we found a significant correlation between the expression of ESR1 and SYNE1, ESR1 and PGR and TNF and SYNE1 in samples taken during the follicular phase of the menstrual cycle.

## Conclusions

Our results show that SNPs rs9371601 and rs3093664 in the SYNE1 and TNF genes respectively, are associated with MM. The present study also provides strong evidence to support the correlation of ESR1, PGR, SYNE1 and TNF gene expression in MM.

